# Working memory and decision making in children with ADHD: an analysis of delay discounting with the use of the dual-task paradigm

**DOI:** 10.1186/s12888-020-02677-y

**Published:** 2020-06-01

**Authors:** Rosa Angela Fabio, Marilla Bianco, Tindara Caprì, Flavia Marino, Liliana Ruta, David Vagni, Giovanni Pioggia

**Affiliations:** 1 Department of Experimental and Clinical Medicine, University of Messina, Via C. Valeria, 98125 Messina, Italy; 2 Italian National Institute of Health, Rome, Italy; 3 CNR-IRIB, Institute for Biomedical Research and Innovation, National Research Council of Italy, via Torre Bianca, Mortelle, Messina, Italy

**Keywords:** ADHD, Cognitive load, Decision-making, Delayed discounting, Working memory

## Abstract

**Background:**

Deficits in working memory tasks have been widely documented in Attention Deficit Hyperactivity Disorder (ADHD) studies. The aim of this study is to evaluate the effects of working memory load in impulsivity during decision-making processes. A delayed discounting (DD) paradigm was used, comparing children with ADHD and age-matched controls.

**Method:**

Thirty-two children equally divided between typically developing and ADHD, from 8 to 10 years of age were assigned to sessions of a dual-task paradigm. In the primary task the child has to choose between two different amounts of money at different time delays, while in the secondary task the child has to repeat a random series of digits with different lengths. The experiment was conducted in a school setting.

**Results:**

Compared to peers with typical development, delayed discounting was significantly stronger in children with ADHD and discounting rates increased in both groups for heavier memory loads. Furthermore, the memory load impact on frequency of immediate rewards was stronger in children with ADHD compared to typically developing children.

**Discussion:**

Results are discussed in terms of the relation between working memory load and decision-making processes, their impact on impulsive behaviour in ADHD and the need for future research to understand possible neurocognitive correlates and use that information to develop better inclusive policies.

## Background

Delayed Discounting (DD) refers to the human preference for smaller but quicker rewards rather than larger but delayed rewards [1] due to the subjective devaluation of rewards as a function of the delay to their delivery.

Children with ADHD are characterized by elevated inattention, hyperactivity, and impulsivity [2], and more generally present differences in executive functioning, comprising an alteration of the reward processing networks [3] and choice impulsivity [4]. Therefore, they are well suited to study the relations among neurodevelopment, delayed discounting and the larger executive functioning network subsiding decision making.

### Delayed discounting and decision making

The process of decision-making is based on the choice between alternative behaviours. Psychological and economic studies consider ways in which gain, losses and probabilities are associated and combined to generate informed choices. In such studies, the attention has been focused on determining whether, in choices that may involve a short-term sacrifice for a long-term gain, the promptness with which a reward is obtained is perceived as an important factor in the process of decision-making [5–10]. Delayed Discounting (DD) paradigms are the most used to study the process leading to a choice. DD tasks involve a series of choices between receiving a small but faster (usually immediate) reward or a larger but delayed reward (DR) [11].

The DD task, in which one chooses between sooner/smaller or later/larger rewards, has proven useful in revealing deficits in executive functioning in various clinical groups [12, 13]. Individual differences that are likely to occur in DD explain important functional differences in decision-making. For example, some people are likely to engage in temporary “myopic decisions” defined as the inability of individuals to realise that their action might implicate consequences [14, 15]. Such decisions facilitate immediate choices but are impulsive and suboptimal in in the long-term, as the immediate results they lead to are overestimated compared to those deferred.

Most studies using the DD paradigm characterize an individual’s choice by generating a discount function that models the effect of delay on subjective value of later rewards [16, 17]. The subjective value *V* of a reward can be estimated using the present value *A* of the delayed reward and is described mathematically as a hyperbolic curve with the following equation:

$V=\frac{A}{1+k\cdotp D}$ where *D* is the delay in the delivery of the reward, and *k* is a free parameter that describes the rate of discounting [15]. Smaller values of *k* indicate a lack of discounting and a preference for delayed rewards, while higher values indicate strong discounting and a preference for immediate rewards.

Higher rates of DD are found in subjects who are willing to decline greater rewards available in the near future, and that show a preference for smaller rewards available immediately [17]. Greater willingness to wait for larger but delayed rewards (usually the indicator of a lower DD), has been associated with less impulsivity [18], better cognition and executive function [19, 20]. Thus, higher values of *k* are indicative of high levels of impulsivity [21].

### Delayed discounting and working memory

Some aspects of the cognitive components of temporarily myopic decisions can be explained in terms of working memory (WM) [18, 22, 23]. WM is a brain system that supports temporary storage and manipulation of information necessary for complex cognitive tasks, such as language comprehension, learning and reasoning [24, 25].

The multi-component model of WM theory proposes a hierarchical structure with three slave subsystems to store and manipulate information and contains a temporary representation of the flow of information into and out of memory [26]. The three components are the phonological loop, the visuospatial sketchpad and the episodic buffer. The phonological loop stores phonological information (the sound of language) and prevents its decay by continuously refreshing it in a rehearsal loop. The visuospatial sketchpad stores visual and spatial information. Finally, the episodic buffer holds representations that integrate phonological, visual, and spatial information, and possibly information not covered by the slave systems (e.g., semantic information, musical information). The episodic buffer is also the link between working memory and long-term memory, with possible implication for ADHD symptomatology [22, 27, 28].

The master subsystem is the Executive Working Memory (EWM) and it functions as a control centre, directing information between phonological, visuospatial and episodic components. The central executive is also responsible for: (1) directing attention to relevant information; (2) shift attention between information used to perform tasks of planning and decision making; (3) suppressing irrelevant information and inappropriate actions and (4) coordinates cognitive processes when more than one task is carried out at the same time [24].

Optimal choice between two or more alternatives involves greater EWM capacity, because the subject should: (1) shift attention between the different options; (2) keep in mind short- and long-term goals; (3) inhibit distraction from decision-irrelevant information; (3) weight the options through the assessment of costs and benefits; (4) access long-term memory for knowledge aiding the decision. Therefore, EWM can be studied through tasks monitoring the completion of goal-directed actions during distractions and dual task paradigms [29].

A few previous studies showed evidence for the effect of WM load on DD, where a decreased WM capacity increases impulsive decision-making [30, 31]. We were able to find only two studies exploring this effect in clinical populations. In adult participants with externalizing psychopathologies, WM load increased discounting rates and WM capacity was significantly associated with higher discounting rates also when controlling for intelligence quotient (IQ), but only after a WM load [32]. In the second study the WM load increased discounting in control participants but not in Alcohol use disorder (AUD) participants [33].

### ADHD and discounting

Previous studies have demonstrated a developmental progression of DD: children show very high values of *k.* Instead, adults are more tolerant with delays and show relatively low *k* values [34, 35]. However, children and adults with ADHD may show even higher indices of DD. Garon, Waschbüsch and Moore [36] showed that decision making in people with ADHD is less effective than that in people without ADHD. People with ADHD may lack focus in decision-making processes, and this can lead them to elect suboptimal alternatives [5, 37–39].

A recent systematic review [40] selected 5 studies with children with ADHD (*n* = 231) and found contrasting evidence for DD in ADHD. Only three of five studies found greater temporal reward discounting for ADHD children. An age and IQ effect on DD was reported for most studies with younger ages and lower intelligence associated with greater discounting. Four of the studies [41–44] used, as the present one, a computerized task with hypothetical rewards.

The first two studies used long delays (7 to 180 days). Antonini and colleagues [41] reported no significant differences among children with ADHD, ADHD plus Oppositional Defiant Disorder (ODD), and those with typical development (TD). However, there was a moderate effect size in the comparison of ADHD children and controls (*d* = .332). According to Dias and colleagues [42], ADHD-C discount value significantly more than controls (*d* = .442). Nevertheless, in the comparison analysis in correlation analysis controlling age and gender and correcting for multiple comparisons, only the correlations with the AUC measures remained significant, considering the diagnoses (*d* = .327). Authors also used functional Magnetic Resonance Imaging (fMRI) to divide children into three subgroups, according to the connectivity pattern. It was observed that in one of the subgroups, children with ADHD performed significantly worse than controls, and when compared to children with ADHD from one of the other two subgroups. Authors also described a strong correlation between DD gradient *ln(k)* and Area Under the Curve (AUC) with the connectivity from the nucleus accumbens (NAcc) to the Prefrontal Cortex (PFC), including the ventromedial PFC and the left anterior PFC.

The other two studies used a short range of delays (0.5 to 60 s). The first [43] found greater TD in the ADHD group than in the control group. However, this difference occurred specifically for girls and in a condition with real rewards and delays (*d* = .480). The second study [44] observed a greater TD in children with ADHD when compared to typically developing children (*η*^*2*^_*partial*_ = .092) but only in the condition with hypothetical rewards and real delays.

A possible interaction effect between DD and ADHD could be the role of working memory. Previous studies have shown that WM deficits are associated with ADHD and impulsivity [45–48]. For example, individuals with low WM capacity are more susceptible to increased impulsive behaviour due to a lower capacity of working memory to modulate response inhibition [49]. Moreover, impulsive behaviour reflects a deficit in inhibition of control of immediate behaviour, planning and evaluation of future options [50].

To our knowledge there is only one study comparing ADHD and TD on DD task with cognitive loads [4] using a Go-no-go task on a real time DD. Martinelli and collaborators found that the effect of cognitive load on response control was associated with greater discounting for children with ADHD, but not for control children. There are no studies in the literature using either a memory load task or hypothetical conditions with cognitive loads.

### Aims of the study

In agreement with the results of Hinson, Jameson and Whitney [30] this study analyses the association between the executive control system and impulsivity [24]. Moreover, it will consider the influence of cognitive load on DD. Different working memory loads will be used in a dual-task paradigm to determine the effect of the WM in the processes of thought and language [51, 52]. In a typical dual-task, participants will be asked to keep a series of numbers or letters in mind while performing the task of primary interest. This load occupies the phonological loop and, also, it disrupts the attentional resources of the EWM [25]. The manipulation of memory load will be used to interfere directly with the WM and to determine whether this interference has an impact on performance in the DD. The performance of the primary task (i.e. decision making) with the secondary task will be compared with a control condition that has a similar response to requests but does not require the maintenance of information in WM.

Previous studies showed contrasting results for DD tasks in children with ADHD, especially when the task involves hypothetical rewards. Only few studies have researched the impact of memory load on DD and showed different pattern of results in typical and clinical populations. To our knowledge there were no studies investigating the effect of WM load in children with typical development or ADHD.

Therefore, the hypotheses of this study are as follows: (1) Children with ADHD in the Experimental Group (EG) will find more difficult than Control Group (CG) to defer a reward; (2) once the memory load increases the deferment of rewards becomes increasingly hard for all participants (both EG and CG) and (3) the increase of memory load have a larger impact on the performance of EG than CG.

## Methods

### Participants

#### Inclusion criteria

Inclusion criteria for all participants were as follows: (1) between 8 and 10 years of age; (2) have a score above 70 in the verbal and performance IQ on the Wechsler Intelligence Scale for Children - IV Edition (WISC-IV); and (3) have no history of brain damage, epilepsy, psychosis, autism spectrum disorder (ASD), bipolar disorders (early-onset bipolar disorder), Tourette’s syndrome, childhood depression; (4) have no current aggressive behaviour or severe oppositional tendency; (5) have no hearing, visual, or physical disabilities, and (6) not being under psychiatric medication.

Children with comorbid disorders were excluded to increase the specificity of the results and avoid confounders. More specifically, children with ODD can be uncooperative, hostile toward authority and behave with the aim of annoying others. Including children with ODD in the study could have raised the doubt that a lower score could have been due to uncooperativeness or plain opposition to the task instead of a neurocognitive deficit.

The hypothetical monetary DD task has been used extensively with adolescents and occasionally with children. However, a recent study found that the generated data suggest a disengagement or misunderstanding of the task [53]. Thus, to ensure the understanding of the task, we required all participants to show good understanding of the concepts of time and money using the Concepts of Time and Money Questionnaire (CTMQ).

Participants included in the EG also have a cut-off severity scores of 14 or higher in both ADHD-I (inattentive subgroup) and ADHD-H (hyperactive subgroups) subscales, i.e. ADHD-C criteria (combined inattentive and hyperactive subgroups) on the Italian versions of the ADHD Rating Scale for Teachers (SDAI) [54, 55] and a clinical diagnosis of ADHD based on the Diagnostic and Statistical Manual of Mental Disorders (DSM-5) [2] criteria obtained from a licensed clinical child psychiatrist. The diagnosis was further confirmed through an additional assessment by the consensus of experienced clinicians in the research team (i.e. child psychiatrists and clinical psychologists).

#### Recruited population

*N* = 414 Italian children, aged 8 to 10 years old, attending 4th or 5th grade of twelve public primary schools in Lombardy, Northern Italy, were recruited. Referrals for children with ADHD were obtained from professionals taking part in an ongoing research and placement training program. All contacted schools agreed to participate in the research and were located in urban areas with an average socio-economic status (SES), private schools were not included. A first screening, administered by teachers, based on SDAI and the Disruptive Behavior Disorders Rating Scale (DBDRS) [56] were used to assess ADHD traits in the participants prior to the beginning of the study.

Teachers were asked to observe the recruited children for 2 weeks and to complete SDAI and DBDRS for each child. Subsequently, they had to report the frequency of any problematic behaviour according to a Likert scale from 0 (problematic behaviour never presents) to 3 scores (very often present) for each questionnaire.

Thirty children were eligible for the EG according to the ADHD-C criteria on SDAI and were further assessed using CTMQ. Each participant was asked to answer 16 open-ended questions on knowledge regarding the values of money and time. Based on the results, *N* = 22 children were eligible for the EG.

**Fig. 1:**
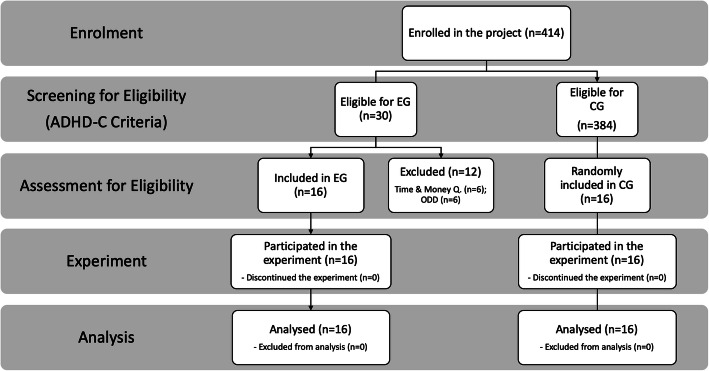
Subjects Recruitment, Assignment, and Assessment Procedures

WISC-IV [57], DBDRS and the Parent Interview for children Symptoms (PICS-IV) scales were administered to the children eligible after CTMQ. These scales aimed to identify disruptive behaviour disorders or other psychiatric disorders, together with getting information about school achievement from parents, teachers, and students themselves. After this test, *N* = 6 children were excluded because diagnosed with ODDs (Fig. 1).

The digit span of each of the participants was assessed before starting the experiment using the digit span forward in the WISC-IV. Assessments’ data for each participant are reported in the Supplementary Materials, sheet S1.

The selected children were individually examined by experienced professionals of the research team and parents were interviewed to confirm their authorisation to be part of the EG.

*N* = 16 children (12 M:4F; mean age in years = 8.75; SD = .48) fully met the inclusion criteria and were enrolled as EG in the present study.

In the final sample there were 14 classes from 11 schools with either 1 or 2 children for each of the twelve participating classes in each group.

The selection was performed separately for each participating school and class to ensure the best matching among social and educational exposure and experimental setting.

We used a randomized block selection among children eligible for the CG to ensure that the groups were balanced for number of children, class, and gender. *N* = 16 children (12 M:4F; mean age in years = 8.62; SD = .62) were randomly assigned to the CG.

All selected children for CG participated in the study and were assessed using the same method used for the EG. All children in the CG meet the inclusion criteria. We had no dropouts.

The EG displayed significantly higher scores than CG on both SDAI hyperactivity, *t*(22.4) = 38.5, *p* < .001, SDAI inattention subscales, *t*(15.0) = 70.5, *p* < .001, while there were no significant statistical differences in IQ, *t*(30) = 1.29, *p* = .208 and Digit Span Forward task, *t*(30) = .582, *p* = .565. Furthermore, there were no difference in time and money understanding on CTMQ: Time scale, *t*(28.7) = 1.17, *p* = .253, Money scale, *t*(22.6) = 1.85, *p* = .077.

The Control Group was comparable to the EG regarding IQ, age, year of education, Digit Span and CTMQ scores. All children in the CG showed no clinical signs of ADHD or other developmental conditions.

Demographic and assessment data for both experimental and control groups are summarised in Table 1.

**Table 1:** Demographic Characteristics and Assessment of the Sample

	ADHD Group (***n*** = 16; M:F = 12:4)			Control Group (***n*** = 16; M:F = 12:4)			Comparison between Groups		
Variable	Mean	SD	Full Range	Mean	SD	Full Range	***t***	***df***	***p***
Age (years)	8.75	.45	8–9	8.63	.62	8–10	.655	30.0	.518
IQ	99.8	6.3	93–110	102.2	4.2	96–113	1.29	30.0	.208
SDAI-H*	16.0	1.5	14–19	.188	.750	0–3	38.5	22.4	<.001**
SDAI-I*	16.7	.9	15–18	.000	.000	0–0	70.5	15.0	<.001**
Time*	15.6	.5	15–16	15.8	.4	15–16	1.17	28.7	.252
Money*	15.7	.5	15–16	15.9	.3	15–16	1.19	22.6	.077
Memory Span	5.56	1.03	4–8	5.57	.78	5–7	.582	30.0	.565
Half Memory Load	2.63	.61	2–4	2.63	.50	2–3	.000	30,0	1.00

* Equal Variance Not Assumed, Signifcant Levene’s Test of Equality of Variances

** The different is significant with alpha < .05

### Measures

The SDAI is an ADHD questionnaire widely used in Italy, validated and standardized for the Italian population [58, 59]. It is composed of 18 items matching the symptom domain of ADHD as described in the DSM-5 [2]. It has a reliability of 0.80 (Inattentive subscale) and 0.74 (Hyperactive/Impulsive), optimal discriminatory power and concurrent validity (*r* > 0.95 [55];. Its test-–retest reliability is 0.83 and 0.81) for inattentive and hyperactive/impulsive respectively [60].

The DBDRS is a 45-question screening measure, completed by either parents or teachers, designed to identify symptoms of ADHD, ODD, and conduct disorder in children and adolescents.

The CTMQ was developed for this study and is reported in the supplementary materials. The cut-off was set to 15 correct answers among 16 questions in both time and money scales. CTMQ was based on our knowledge of children’s cognitive development and reviewed by a focus group of five primary school teachers. The majority of students are expected to pass the test and it is designed to assess the skills’ level needed to correctly understand the experimental task. CMTQ was included in the study because children with ADHD can have difficulties with magnitude-related concepts like space, time and numbers [61]. Researching the relation between those deficits it’s outside the scope of our experiment but previous studies shown a neurological link with the implication of the parietal cortex in both magnitude representation [62] and ADHD [63]. In our study, which focuses on impulsivity and decision making, possible difficulties in time and monetary value representation would be confounders.

### Experimental design

A dedicated application was developed for the administration of the individual sessions. All sessions were administered in a quiet classroom of the school, equipped with computers, monitors, chairs and desks. All participants were asked to sit in front of a monitor in order to complete the tasks. Each session consisted of one preliminary trial and 3 blocks with different memory loads with 16 experimental trials for each block comprising the random combination of 4 delay and 4 reward conditions. Each session lasted 20 min.

#### Preliminary task

Each of the *N* = 32 participant was asked to engage in a second individual assessment of memory span using the software developed to run the experiment instead of the WISC-IV [57]. The aim of this preliminary task was to find the baseline digit span for the participant given the different experimental condition between the experimental setting and the initial assessment.

For the preliminary assessment of maximum memory load, the first sequence was 3-digits long. The number of digits to repeat would increase if the participants successfully managed to repeat all of them. A new number sequence was randomly generated before each trial.

#### Memory load task

In the memory load task, the participant was required to listen and repeat the assigned sequence of random numbers.

Three different conditions were tested and counterbalanced among participants: (1) delayed reward option task without the memory load task; (2) dual-task with the delayed reward option task and the memory load task with half memory load and (3) dual task with full memory load.

The initial full load was set to the maximum digit span assessed in the preliminary task minus one (*n*-1), while the half load was set to *n*/2 if *n* was even and (*n*-1)/2 if *n* was odd.

We choose (n-1) as the full load condition to minimize the number of errors while maintaining a high load on WM. This task not only functions as an interference but it’s also a measure of the attention to the experiment. A high number of errors in this task would mean that participants are deploying their attentional resources mainly on the primary task instead of splitting them between the two tasks.

In the conditions with half and full memory load, the random number sequence was presented before the choice task, participants were therefore asked to remember the sequence and repeat it aloud through the choice task. To ensure that the participants’ cognitive resources were focused on the memory load task, if an error in repeating the digits was made, another series of the same digits was presented; if the participant failed also to repeat the second series, the task was stopped and the number of correct digits was recorded as the maximum digit span of the participant for that specific trial.

#### Delayed reward task

The primary task consisted of a series of choices between a small monetary reward obtained after a shorter time and a larger monetary reward obtained after a longer time. The delayed reward option task comprised a series of 16 trials. In each trial, the participant was asked to select between two options e.g. the first option was always a smaller amount of money the same day, whilst the second option was a larger amount of money deferred in different periods of time. Each participant knew that there were no right or wrong choices. Trials comprised 4 possible reward combinations: €1–€11, €2–€12, €3–€13 and €4–€14, with a reward difference always set to €10; and 4 possible time delays of 1 week, 1 month, 6 months and 1 year. An algorithm specifically designed for the test had selected the order of the 16 trials randomly and balanced them among participants.

The full set of responses from the money/time option task and digit spans for different memory load condition are reported in the Supplementary Materials, sheet S2.

### Statistical analysis

All statistical analyses were run through SPSS software (v. 23, IBM Corporation, Armonk, NY, USA). The descriptive statistics of the dependent variables were examined. The dependent variable (the value of *k*) were submitted to repeated measure ANOVA with one between-subject factor (group), and two within-subject factors: (1) three memory load conditions (zero load, half load, full load) and (2) four time delays (1 week, 1 month, 6 months and1 year); trials within the same condition but different monetary rewards were averaged together. The alpha-level was set to .05 for all statistical tests. All effect sizes and post-hoc tests’ power are reported in sheet S3 of Supplementary Materials. The effect sizes were computed using partial eta-squared. Omnibus tests were evaluated with two-tailed alpha-level = .05. Planned pairwise comparisons were performed among groups using *t-*test and ANOVA, alpha-level was Sidak’s corrected. Greenhouse-Geisser correction was used for effects failing the Mauchly’s test of sphericity.

With reference to the primary task parameter, the inverse formula of $V=\frac{A}{1+k\cdotp D}$ was calculated: $k=\frac{A-V}{V\cdotp D}$ . *k* is a parameter that measures the decrease rapidity in subjective value over time. A *k* value of zero shows an absolute preference for delayed choice, while higher *k* values show an increase in DD. In our analysis we used Euros for *A* and *V* and weeks for *D* (setting a month to 4 weeks) as units. The specific units of measurement that have been chosen change the results only by a multiplicative factor, the choice is therefore irrelevant for the purposes of statistical analysis.

Preliminary ANOVA was carried out to examine the secondary task performance, assuming digit span as the dependent variable to ensure that the relevant variable was the memory load and not the memory span. Correlations among variables were calculated to assess their impact on the analysis.

Delayed discounting rate defined as *D*_*R*_ *= −ln(k)* is also commonly reported in the literature, especially when researchers prefer to use logistic regression instead of the inverse formula to compute *k* [64]. Therefore, we used it as a supplementary analysis for comparisons between groups and among different WM loads.

## Results

For both groups of participants, the percentage of errors on the memory load task was 0% in the half load condition and 15% in the full load condition.

The preliminary analysis revealed no effects of the maximum digit span on the primary task results (see sheet S4 of Supplementary Materials).

Correlation among SDAI and DBRDS subscales was very high (.906 to .988) and also their correlation with outcome variable *k* and *ln(k)* for different WM load conditions (*r* = .744 to *r* = .917, *p* < .001). Outcome variables among different WM load conditions were highly correlated, *k* (*r* = .599 to *r* = .778, *p* < .001) and *ln(k)* (*r* = .758 to *r* = .882. *p* < .001). Furthermore, IQ was correlated with CTMQ-Time (*r* = .544, *p =* .001) and Digit Span was correlated with CTMQ-Money (*r =* .445, *p* = .011). IQ, age and CTMQ were not correlated with outcome variables (*p >* .155) with the exception of a trend between IQ and *k* in the zero WM load condition (*r* = −.314, *p* = .080) and CMTQ-Money and *k* in the zero WM load condition (*r* = −.321, *p* = .073).

In the primary multivariate analysis, both memory load, *F* (2,29) = 33.8, *p* < .001, *η*^2^_*p*_ = .700, and time delay, *F* (3,28) = 110, *p* < .001, *η*^2^_*p*_ = .922, were significant main effect on *k* in the multivariate test.

**Fig. 2:**
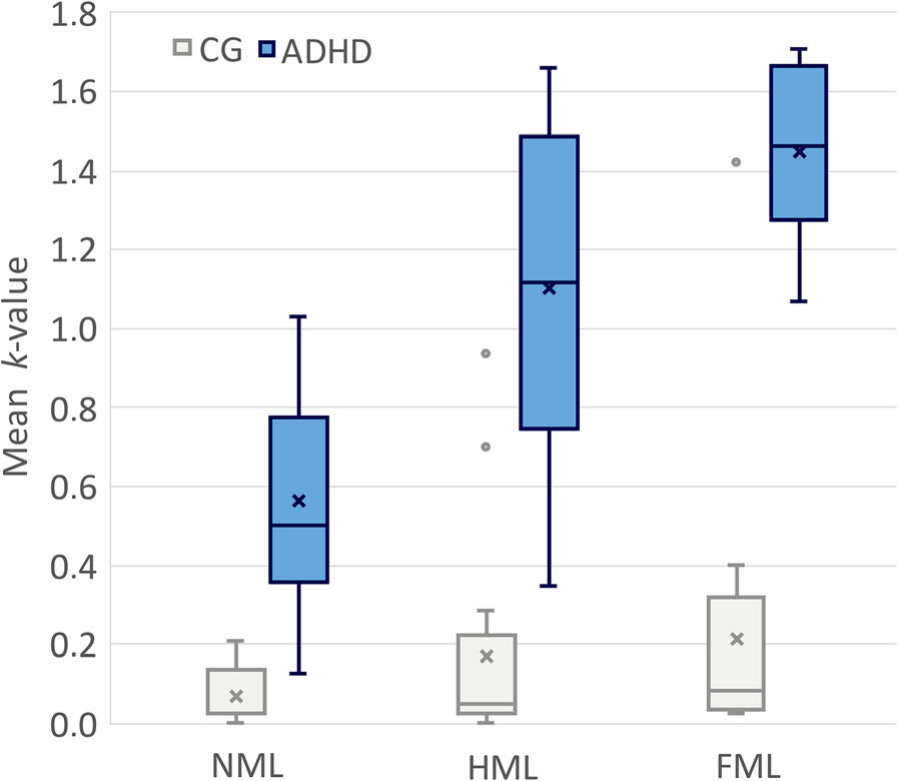
*k-*value Comparison Between Control and Experimental (ADHD) Group for Different Memory Load Conditions. *Footnote*: In the Box and Whisker Plot a box is drawn from the first quartile to the third quartile, while a line is drawn at the median and the cross is the mean value. The whiskers extend from each quartile to the minimum or maximum. Outliers are depicted as dots

ANOVA revealed also a significant main effect of Group *F* (1,30) = 228, *p* < .001, *η*^2^_*p*_ = .884, post-hoc comparison showed that EG > CG, with M = 1.04, S.E. = .041 for EG and M = .150, S.E. = .041 for CG, indicating that the EG showed higher *k* value than CG. Furthermore, a significant Group × Memory Load interaction effect was found, *F* (2,29) = 17.2, *p* < .001, *η*^2^_*p*_ = .543, post-hoc comparison showed that *k* increased more in EG when the memory load was heavier as depicted in Fig. 2.

All the other 2-ways and the 3-ways interactions were also significant. Group ×Delay interaction effect, *F* (3,28) = 75.3, *p* < .001, *η*^2^_*p*_ = .890, post-hoc comparison showed a larger decrease of *k* with time in the EG compared to CG, and a larger difference for shorter reward delays. We also found a Memory Load × Delay interaction effect, *F* (6,25) = 11.8, *p* < .001, *η*^2^_*p*_ = .740. A post-hoc comparison showed a larger effect of memory load for shorter reward delays.

**Fig. 3:**
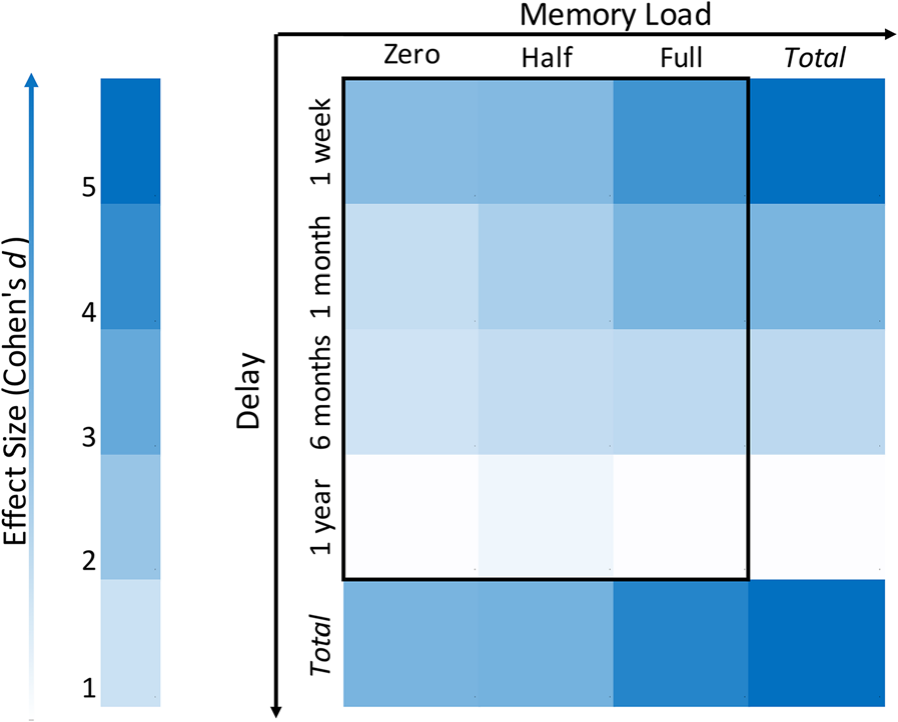
Effect Size Matrix of The Difference Between ADHD and Control Groups for Different Delays and Memory Loads

Finally, we found the 3-ways interaction effect among Memory Load, Delay and Diagnosis, *F* (6,25) = 5.69, *p* = .001, *η*^2^_*p*_ = .577, revealed that the heightened effect of memory load for shorter reward delays was more accentuated for the EG. Group comparison for each condition is reported in Table 2 and the difference in effect size is depicted in Fig. 3. The full analysis is reported in sheet S6 of Supplementary Materials.

**Table 2:** Means and Standard Deviations (SD) of *k* value

***Time***	Groups	Memory Load					
		Zero		Half		Full	
		M	SD	M	SD	M	SD
*1 week*	ADHD	.1.56***	.950	3.41***	1.48	4.36***	.766
	Control	.000	.000	.365	1.01	.365	1.30
*1 month*	ADHD	.485**	.404	.752***	.428	1.16***	.221
	Control	.124	.207	.166	.274	.312	.410
*6 months*	ADHD	.132*	.055	.145**	.045	.172**	.041
	Control	.066	.086	.069	.080	.089	.084
*1 year*	ADHD	.075	.029	.085	.023	.080	.030
	Control	.080	.037	.077	.036	.086	.028

* *p* < .05, ** *p* < .005, *** *p* < .001 using t-test on the differences between groups, equal variance not assumed

The ancillary analysis with *ln*(*k*) as a dependent variable led to a significant within subject effect for memory load, *F* (2,29) = 14.7, *p* < .001, *η*^2^_*p*_ = .509 and between subject effect for group, *F* (1,30) = 114, *p* < .001, *η*^2^_*p*_ = .792. The Group×Memory Load interaction effect was not significant when *ln*(*k*) was used as a dependent variable, *F* (2,29) = .272, *p* < .764, *η*^2^_*p*_ = .018.

To test the robustness of the results we ran two other ancillary analysis: in the first one we used a simple ranking for the delays (1, 2, 3, 4) instead of the number of weeks (1, 4, 26, 52), to approximate a contraction of perceived time for longer delays; in the second one, we used the number of delayed reward responses as the dependent variable instead of *k,* to avoid any theoretical assumption on the shape of the discounting curve. In both analyses all effects were significant and in the same direction as the primary analysis. Hence, the effects are not an artefact of the specific formula used to study DD.

The three ancillary analyses are reported in the supplementary materials in sheets S7, S8 and S9.

Gender differences in ADHD was not one of the aims of the study. However, for future comparisons, it was possible to run the primary analysis excluding female participants.

Results were in line with the ones found for the whole group. ANOVA revealed a significant main effect of Group *F* (1,22) = 163, *p* < .001, *η*^2^_*p*_ = .881, and Group × Memory Load interaction, *F* (2,21) = 9.85, *p* < .001, *η*^2^_*p*_ = .309.

## Discussion

All hypotheses of the study have been confirmed: (1) children with ADHD show higher levels of DD than control subjects, (2) once the memory load increases, deferring a reward becomes harder for both children with ADHD and with typical development, and (3) the performances of children with ADHD are significantly worsened by the addition of a memory load compared to the control ones.

**Fig. 4:**
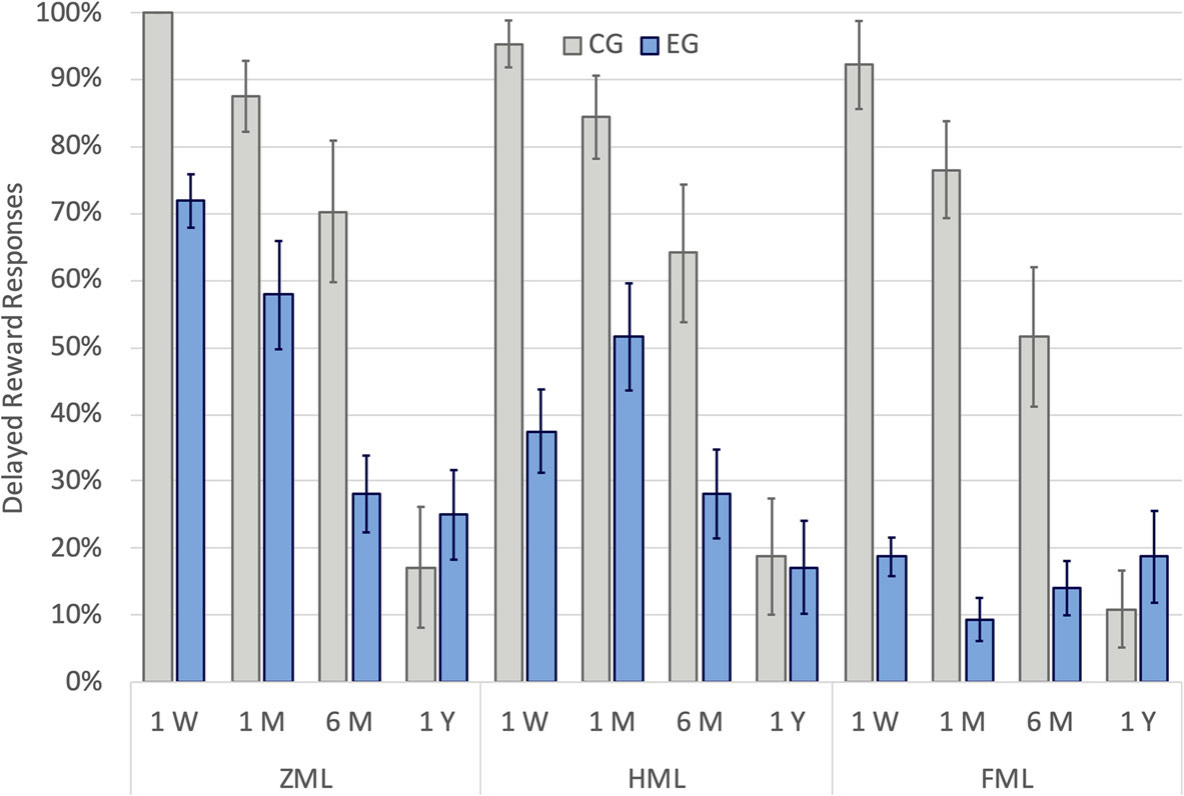
*k-*value Comparison of Choose Delayed Reward Between Control and Experimental (ADHD) Group for Different Conditions. *Footnote*: Error bars are standard errors. All differences between the two groups are significant (*p* < .05) with the exception of the three 1-year conditions. A table with the plot-data and the statistical analysis is reported in Supplementary Materials in Table S.10

For the sake of clarity, we will discuss the results based on proportion of delayed reward, for each group and each condition. Data is reported in supplementary materials in sheet S10 and is depicted in Fig. 4.

In almost all conditions we have a decrease in delayed rewards responses with the increase of time delays, with a few exceptions that are not statistically significant:

1. in all conditions and for both groups: ZML > HML > FML
2. In all conditions: EG < CG, with the exception of 1-year delay.

For the 1-year delay EG and CG are comparable, but in the CG there was (graphically) a huge change in the discounting slope. Wittmann and colleagues [65, 66] have previously shown that the discounting slope changes for delays shorter and larger than 1 year. Furthermore, the different slopes before and after the 1-year cut-off, correlate with a differential activation of the caudate nucleus and putamen in typical adults. A similar mechanism could be in place in our study and therefore the mechanism involved in the 1-year delay could be different and have different maturational timing.

The decrease in group differences between high impulsivity (ADHD-C) and low impulsivity (TD) groups for increasing time delays, also suggest the presence of a non-linear scaling, as expected using hyperbolic delayed reward discounting curves that take into account the contraction of time for future events [67].

The analysis of the results also found that the number of delayed choices decreases in ratio with the increasing of time gap between immediate and delayed options. This data confirms that the value that the individual attributes to the different choices decreases as the reward becomes more distant in time [11, 68].

The findings of this experiment are consistent with the literature of the discounting parameter *k*, influenced by the chosen behaviours of the subject. As Green and colleagues [11, 68] stated, impulsivity pushes the individual to make suboptimal choices, as immediate rewards are overvalued compared to those deferred. At the moment of choosing, subjects with WM deficits [69] or a higher memory load carry out inadequate choices and *k* value increases.

Two of the previous studies found evidence of a larger DD in ADHD compared to typical development only for real delays. Nevertheless, both studies employed delays of less than 60 s, and we suggest that the time frame was too short to elicit and impulsive behaviour during a completely hypothetical task. The other two studies employed longer delays and found evidence in the same direction as our study, but in those studies the evidence was less strong (*d* = .302 for Antonini and colleagues, and *d* = .442 for Dias and colleagues, but did not survive correction for multiple comparisons, *p* = .054). In contrast, for the condition without memory load, we found a very large effect size, *d* = 2.59. The smaller sample size could have inflated our results, thus caution in interpretation of our data is mandatory. However, a careful examination of the data (Fig. 4) shows that the presence of impulsive choices in the control group is very low (0% in the 1-week condition) while in the ADHD group it was already high (28.1% in the 1-week condition) and without outliers. A comparison between the zero-load condition of our study and a similar condition in the study of Wilson and colleagues [70] shows that our unexpectedly strong results are mainly led by a higher discounting for the ADHD group (a possible explanation is discussed at the end of the conclusions).

A significant finding in this study is that a WM load, designed to deplete EWM capacity, substantially increased delay discounting rates for all subjects. The current study extends the work of Finn and colleagues [32] on adults with and without high Externalizing Behaviours, to children with and without ADHD. Our results mimic the findings of previous studies where the discounting rates *ln(k)* does not vary differently between the experimental and the control group. Nevertheless, we found a greater effect of a WM loading ADHD compared to control for both *k* and the proportion of delayed choices.

### Limitations

We chose a restricted range of age (8 to 10 years old) and IQ within the normal range, to avoid the confounding effect of intellectual ability and maturational processes, that choice impacts on the generalisability of the study. Moreover, although the selection process adopted by this study made possible to create a detailed assessment of the participants, it reduced considerably the number of final participants, lowering the power of the study even if the results were statistically significant.

Previous studies suggested differences in symptoms between males and females with ADHD [71, 72] with increased DD among females but not males [43, 72]. We had only 4 females for each group in our final sample. Therefore, the study was underpowered to detect gender differences thus we didn’t include them in the analyses. Nevertheless, we excluded female participants and obtained for the male-only sample the same results as the initial sample.

The selection process was performed sequentially, therefore we cannot compare children who failed the screening tests (e.g. CMTQ) with children who passed them on outcome measures. Moreover, comorbidities are common in neurodevelopmental disorders, and our choice to exclude children with comorbidities further reduced the generalisability of the results. Nevertheless, we are confident that our choices increased the putative validity of the link between the experimental results and executive functioning circuitry specific to the condition.

A school setting has been chosen for this study for practical reasons and the children had to leave the class to do the assessment. Thus, the level of cognitive resources available to the child while performing the tasks cannot be assured. In addition, as the children were from different schools and there were different examiners, the settings where the assessments were carried out and the examiners that assessed the children differed. That might have reduced the controllability of the experiment. However, it could have increased its ecological validity. Furthermore, for each school/setting the number of children in the EG and CG were the same, therefore confounding factors linked to the specific setting should be negligible in the group comparison.

Finally, one limitation of this study is given by the specific formula used to compute the parameter *k*, which is supposed to be a constant, and that has been found to vary in relation to different temporal intervals. Meaning that the equation chosen to define DD might be too simplistic. However, the supplementary analysis employed to double-check the results using different formulae showed that the behavioural results are robust enough to remain statistically significant regardless of the specific equation used to compute discounting.

## Conclusions

The present study is the first to show the impact of WM on DD in children with ADHD. Moreover, even if the results are statistically significant, the small sample size of the study reduces their strength. Therefore, the study could be seen as an initial step towards more definitive evidence to investigate further the four implications described below.

First, given the same experimental condition, *k* should be a constant, but the results we presented showed a variability of *k* with time. According to Body, Bradshaw and Szabadi [73] time delays have a non-linear scaling following a power law that takes into account the contraction of perceived time. Therefore, future research should aim to create a larger dataset and find an empirical formula aimed to describe *k* in a more precise way using generalized hyperbolic functions or other more sophisticated models instead of the standard hyperbolic discounting. A more complex model, that also includes the many intervening variables, could lead to a richer explanation of decision making in typical development and ADHD.

Second, under the conditions of increased memory load, both the ADHD and the control group reported higher discounting. Therefore, in line with already discussed evidence highlighting the hierarchical structure of the executive system we found a link between the executive working memory and inhibition of impulsive behaviour [30]. Taxing the phonological loop (a slave system) increases the load on the central executive (the master system) subsequently reducing its availability to coordinate the inhibition of impulsive choices.

Third, as expected, the ADHD-C group showed significantly higher levels of *k* than their peers even if they have the same IQ, WM capacity and WM load. Thus, we suggest that even if WM has a significant role in the decision-making and DD processes and reducing its capacity lead to choice impulsivity in children with ADHD, it could not be the solely responsible for the behaviour. Therefore, this paper invites future research to focus not only on replicating those findings, but to expand them, exploring the different dimensions of specific ADHD characteristic and comorbidities, possible differences in the mechanisms involved underlying DD under different ranges of delays, and their maturational changes during development.

Fourth, despite the small range in variability among the demographic variables, we found a correlation between IQ and CTMQ-Time and between Digit Span and CTMQ-Money. Furthermore, we also found a trend for a correlation between IQ and *k* in the zero WM load condition and CMTQ-Money and *k* in the zero WM load condition. Those results invite future researchers to design experiments to better understand, and control for, the influence of conceptual understanding of time and monetary value in DD research.

Fifth, contrary to some of the previous studies [43, 72] reporting DD only in female participants, we found an increased DD in male children with ADHD. We used hypothetical classic DD with longer delays and higher monetary rewards and Digit Span as a cognitive control task, while Rosch, and Mostofsky [43] used shorter delays and Patros et al. [72] used both classic and real-time DD as primary tasks and Go/No-Go, Spatial Span and Stop Signal as cognitive control tasks. We suggest for future research to focus on task × gender interaction in order to shed a light on the phenotypic differences among males and females with ADHD.

Finally, EG and CG showed a comparable increase in discounting rate *D*_*R*_ *=* −*ln(k)* with an increase in WM load. Nevertheless, both *k* and the raw number of impulsive choices, increased more in ADHD than in typical children; for instance, the frequency of choices for immediate reward, in a 1 month delay condition without WM load, was 42% in ADHD and 12% in typical children, while in the full WM load condition, it was 90% in ADHD and 23% in controls. Based on the assumption of hyperbolic discounting and Fechner’s logarithmic psychological function of perception, we can assume that *D*_*R*_, a logarithmic function of the discounting coefficient, is proportional to the perceived delay [74]. Therefore, our result suggests that WM load impacts similarly the two groups on the perceived delay, but given the higher base discounting in EG, the same load will produce larger increase in the number of impulsive choices in children with ADHD.

Taken together, these results suggest that those with ADHD, who already have elevated discounting rates and patterns of disinhibited decision-making, are very vulnerable to the effects of conditions which compromise EWM capacity, such as stress, emotional arousal, or high cognitive load, conditions that are frequently found in a school setting. Under such conditions, those with ADHD may be more likely to engage in impulsive /risky decisions that have significant negative consequences.

This is the first DD experiment with ADHD children run in a school setting. Children were briefly taken from their class after hours of school that already taxed their executive control. Therefore, we can suggest that the larger effect size we found could be related to the specific setting. The behavioural and attentional request made in school, could deplete executive resources in children with ADHD, decreasing their capacity to learn and to inhibit impulsive behaviours. Future research should try to directly compare settings (laboratory vs. school) and fatigue (well rested vs. late hours). Moreover, a systematic assessment of different aspects of the WM system and of the role of different strategies used in task completion for different kinds of cognitive loads, would help tease apart the different factors that affect discounting rates / impulsive decision-making. If the results will be corroborated, and the setting will play an important role in the effect size of the results, they will also be informative for decisional bodies to develop better inclusive policies.

## Supplementary information

**Additional file 1 Table S1.1** Complete Participants’ Demographic Data and Assessment Scores. **Table S2** Complete Experimental Data. **Table S3.1** Multivariate Test, *k* in weeks. **Table S3.2** Mauchly’s Test of Sphericity. **Table S3.3** Tests of Within-Subjects Effects and Between-Subjects Effect, *k* in weeks. **Table S3.4** Pairwise Comparisons, *k* in weeks. **Table S3.5** Parameter Estimates, *k* in weeks. **Table S4.** Tests of Between-Subjects Effects, Preliminary ANOVA using Digit Span as a Covariate. **Table S5.** Correlation among Demographic and Outcome Variables. **Table S6.** Group Comparison among Different Conditions. **Table S7.1** Multivariate Test, *k* computed using ranked delays. **Table S7.2** Mauchly’s Test of Sphericity, *k* computed using ranked delays. **Table S7.3** Tests of Within-Subjects Effects and Between-Subjects Effect, k computed using ranked delays. **Table S8.1** Multivariate Test, Using Number of Delayed Choices as a Dependent Variable. **Table S8.2** Mauchly’s Test of Sphericity, Using Number of Delayed Choices as a Dependent Variable. **Table S8.3** Tests of Within-Subjects Effects and Between-Subjects Effect, Using Number of Delayed Choices as a Dependent Variable. **Table S9.1** Multivariate Test, Using *ln(k)* as a Dependent Variable. **Table S9.2** Mauchly’s Test of Sphericity, Using *ln(k)* as a Dependent Variable. Table S9.3 Tests of Within-Subjects Effects and Between-Subjects Effect, Using *ln(k)* as a Dependent Variable. **Table S10.** Group Comparison of Delayed Choice among Different Conditions.

**Additional file 2.**

## Data Availability

All data generated or analysed during this study are included in this published article and its supplementary information files.

## Abbreviations

ADHD: Attention Deficit Hyperactivity Disorder
AUD: Alcohol Use Disorder
AUC: Area Under the Curve
ADHD-C: Attention Deficit Hyperactivity Disorder - Combined Subtype
ADHD-H: Attention Deficit Hyperactivity Disorder - Hyperactive Subtype
ADHD-I: Attention Deficit Hyperactivity Disorder - Inattentive Subtype
CTMQ: Concepts of Time and Money Questionnaire
CG: Control Group
DD: Delayed Discounting
DR: Delayed Reward
DSM-5: Diagnostic and Statistical Manual of Mental Disorders
DBDRS: Disruptive Behavior Disorders Rating Scale
EWM: Executive Working Memory
EG: Experimental Group
FML: Full Memory Load
fMRI: Functional Magnetic Resonance Imaging
HML: Half Memory Load
IQ: Intelligence Quotient
SDAI: Italian ADHD Rating Scale for Teachers
NAcc: Nucleus Accumbens
ODD: Oppositional Defiant Disorder
PICS-IV: Parent Interview for children Symptoms IV Edition
PFC: Prefrontal Cortex
SES: Socio-Economic Status
TD: Typical Development
WISC-IV: Wechsler Intelligence Scale for Children - IV Edition
WM: Working Memory
ZML: Zero Memory Load
