# Supplementary material for: Working memory and decision making in children with ADHD: an analysis of delay discounting with the use of the dual-task paradigm
Source: BMC Psychiatry. 2020 Jun 1;20:272. doi: 10.1186/s12888-020-02677-y (PMC7268601; doi:10.1186/s12888-020-02677-y)
Supplement: Supplementary file 2 — Additional file 2. [file 12888_2020_2677_MOESM2_ESM.docx]

**Concepts of Time and Money Questionnaire**

NAME_______________

**Can you recognise different amounts of money? YES NO**

| 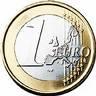 | 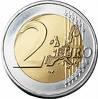 | 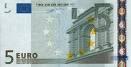 | 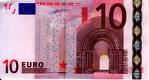 | 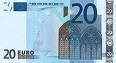 |
| --- | --- | --- | --- | --- |

EQUIVALENCE:

| 3€ | How many 1€ would you need to make 3€? |
| --- | --- |
| 7€ | How many 1€ would you need to make 7€? |
| 10€ | How many 5€ would you need to make 10€? |
| 12€ | How many 2€ would you need to make 12€? |
| 13€ | How many 5€ would you need to make 13€? |
|  | How much is the change? |
| 14€ | How many 2€ would you need to make 14€? |

VALUATION:

| What can you buy with 1€? |  |
| --- | --- |
| What can you buy with 5€? |  |
| What can you buy with 10€? |  |
| What can you buy with 15€? |  |

**Concepts of Time and Money Questionnaire**

NAME______________

Can you read time? YES NO

How long?

| 1 HOUR (in minutes) |  |
| --- | --- |
| 1 WEEK (in days) |  |
| 1 MONTH (in days) |  |
| 1 YEAR (in days) |  |

How long is...?

| 60 MINUTES |  |
| --- | --- |
| 7 DAYS |  |
| 30 DAYS |  |
| 365 DAYS |  |

ESTIMATES

| A TASK IS LONGER IF COMPLETED IN A MONTH OR A WEEK? |  |
| --- | --- |
| WHAT IS LONGED BETWEEN A MONTH AND A YEAR? |  |
| WHAT IS LONGER BETWEEN A MONTH OR A DAY? |  |
| WHAT IS LONGER BETWEEN ONE YEAR OR ONE HOUR? |  |
| HOW LONG DOES IT TAKE TO LAY DOWN THE TABLE? |  |
| HOW LONG DOES IT TAKE TO WALK TO CHINA? |  |
| HOW LONG DOES IT TAKE TO DO A SHOPPING IN THE SUPERMARKET? |  |
| HOW LONG DOES A MOVIE USUALLY LASTS FOR? |  |
